# A Novel and Validated 8-Pyroptosis-Related Genes Based Risk Prediction Model for Diffuse Large B Cell Lymphoma

**DOI:** 10.3390/biom12121835

**Published:** 2022-12-08

**Authors:** Junrui Ma, Wenhan Wang, Jiao Ma, Zizhen Xu

**Affiliations:** 1 Department of Laboratory Medicine, Ruijin Hospital, School of Medicine, Shanghai Jiao Tong University, Shanghai 200025, China; 2 Department of Biochemistry and Molecular Cell Biology, School of Medicine, Shanghai Jiao Tong University, Shanghai 200025, China

**Keywords:** Non-Hodgkin’s Lymphoma, diffuse large B-cell lymphoma, pyroptosis, prognostic prediction model, immune regulatory program

## Abstract

Background: Diffuse large B-cell lymphoma (DLBCL), the most common type of Non-Hodgkin’s Lymphoma (NHL), has a lethal nature. Thus, the establishment of a novel model to predict the prognosis of DLBCL and guide its therapy is an urgency. Meanwhile, pyroptosis is engaged in the progression of DLBCL with further investigations required to reveal the underlying mechanism. Methods: LASSO regression was conducted to establish a risk model based on those PRGs. External datasets, RT-qPCR and IHC images from The Human Protein Alta (HPA) database were utilized to validate the model. ssGSEA was utilized to estimate the score of immune components in DLBCL. Results: A model based on 8 PRGs was established to generate a risk score. Validation of the model confirmed its robust performance. The risk score was associated with advanced clinical stages and shorter overall survivals. Two novel second-line chemotherapies were found to be potential treatments for high-risk patients. The risk score was also found to be correlated with immune components in DLBCL. Conclusion: This novel model can be utilized in clinical practices to predict the prognosis of DLBCL and guide the treatment of patients at high risk, providing an overview of immune regulatory program via pyroptosis in DLBCL.

## 1. Introduction

Diffuse large B-cell lymphoma (DLBCL), an extremely heterogeneous and invasive subtype of Non-Hodgkin’s Lymphoma (NHL), depicts a variety of clinical, pathological and biological phenotypes with distinct genetical variants [1,2]. DLBCL could be subdivided into germinal center B-like DLBCL (GCB), activated B-like DLBCL (ABC) and unclassified via a gene-expression profile [2,3]. Under a standard R-CHOP treatment (rituximab, cyclophosphamide, doxorubicin, vincristine, and prednisone), the overall survival rate of DLBCL can reach 60% [4], while those who have relapsed or are refractory patients, especially after R-CHOP treatment failure, showed a poor prognosis. Although previous studies [2,4,5,6] have identified various biomarkers for the prediction of the prognosis and sensitive drugs in DLBCL, biomarkers with new insights into these mechanisms are required to compensate for the deficiency and limitation of the previous markers due to the heterogeneous and malignant nature of DLBCL. Further, novel biomarkers are also required to anticipate the sensitivity of a novel chemotherapy and thus guide the clinical treatment. There is an urgent call to develop a novel model, from new insights, to evaluate the risk of DLBCL and anticipate drug sensitivity, thus conducting a personalized diagnosis and treatment in clinical practice.

Pyroptosis, triggered by inflammasomes and executed by caspases, refers to one of the programmed cell death (PCD) processes with inflammatory features, which results in the lysis of the cell [7] and the release of the cell’s intracellular immunostimulatory contents [8]. Based on the molecular mechanism of pyroptosis, pyroptotic cell death can be subdivided into canonical pyroptotic death (mediated by inflammasome) [9], non-canonical pyroptosis (with the absence of caspase-4/5) [10], caspase-3/8-mediated pathway and the granzyme-mediated pathway. Generally, pyroptosis, related to both inflammatory process and PCD process, performs both a foe and friend function in cancer progression by its role in inhibiting tumor progression via the PCD process or by accelerating tumor progression via activating growth stimuli [9], which could happen either separately or simultaneously. Although previous studies have recognized that pyroptosis contributed to the initiation, proliferation, invasion, and metastasis of various tumors [11], the role of pyroptosis in DLBCL was still unclear, hence requiring extensive studies to reveal the immune regulatory mechanisms underlining pyroptosis in DLBCL process.

The tumor microenvironment (TME) refers to the tumor cells and their surrounding stromal area, which comprise a spectrum of cell types (such as fibroblasts, immune cells, and stromal cells) and extra-cellular components (such as cytokines, chemokines, and metabolic products) [12]. Immune components in TME have been demonstrated to affect the tumor progression and treatment outcomes in DLBCL [7,13]. Intriguingly, accumulated evidence has indicated that pyroptosis also influences the immunotherapeutic outcome by modulating immune cellular infiltration [7], suggesting that tumor immunity and inflammation are highly correlated to pyroptosis [14]. However, the mechanism underlining how pyroptosis contributes to the DLBCL microenvironmental progression and the immune responses remains unclear. Moreover, the role of pyroptosis-related genes (PRGs) in the DLBCL progression and prognosis and the corresponding regulatory programs are still undetermined. Along with the uncertain function of PRGs in DLBCL, they are still promising to serve as prognostic indicators with regards to their wide involvement in tumor progression [15].

Considering the wide engagement and potential prognostic value of PRGs in the progression of DLBCL, we believe that pyroptosis will give a new insight to establish a risk model. Thus, we established a novel risk prediction model based on pyroptosis related genes to generate a risk score. High-risk scores were found to be associated with advanced clinical stages and a shorter overall survival. The risk score was also found to be correlated with immune components in DLBCL. In vitro and in silico validation confirmed the robust performance of the model. Two novel second-line chemotherapies were found to be potential treatments for high-risk patients. Overall, this model can be utilized in clinical practices to predict the prognosis of DLBCL and guide the treatment of patients at high risk, providing an overview of an immune regulatory program via pyroptosis in DLBCL.

## 2. Materials and Methods

### 2.1. Data Acquisition

The mRNA expression profile and the related clinical data were obtained from Gene Expression Omnibus (GEO) database and The Cancer Genome Atlas (TCGA) database. To minimize the survival bias, samples were carefully chosen as follows: (1) samples with an overall survival greater than 0 and (2) samples with complete survival status information. Under this criteria, 412 samples from GSE10846, 121 samples from GSE4475, and 47 samples from TCGA were selected for further analysis.

The immunohistochemistry slice images of *HTRA1*, *RBBP7*, *NFE2L2*, *SCAF11*, *ABL1*, *PAK2,* and *CPTP* in colorectal cancer, breast cancer, prostate cancer, lung cancer, and Non-Hodgkin’s Lymphoma were obtained from The Human Protein atlas (HPA) database to validate the expression of these genes in real-world tumor tissues. The patient IDs of selected slices are presented in Supplementary Table S5.

We set ‘pyroptosis’ as a keyword in the search engine of GeneCards (https://www.genecards.org/ (accessed on 10 September 2022)). The pyroptosis related genes (PRGs) were then obtained from GeneCards and those non-protein-coding genes were filtered. A total of 388 PRGs were obtained (Supplementary Table S1). Further, the master genes in diffuse large B cell lymphoma (DLBCL) were also obtained from GeneCards.

The gene list of immune components (Supplementary Table S7) was obtained from the Supplementary Materials of article generated by Xiao B et al. [16].

### 2.2. Pathway Enrichment Analysis

The Kyoto Encyclopedia of Genes and Genomes (KEGG) and Gene ontology (GO) annotation was performed via Database for Annotation, Visualization, and Integrated Discovery (DAVID) database (http://david.ncifcrf.gov (accessed on 24 October 2022)). Enriched pathways were selected as FDR < 0.05.

### 2.3. Establishment and Validation of the Risk Prediction Model

First, univariate Cox regression analysis was performed to identify prognostic pyroptosis related genes via the R ‘survival’ package. A total of 24 prognostic pyroptosis related genes were selected as *p* value < 0.001. Then, we utilized a random 80% of GSE10846 as training set to perform Least Absolute Shrinkage and Selection Operator (LASSO) regression via R ‘glmnet’ package. Later, Ridge regression was conduct by the same package and procedure to be compared with the LASSO regression model.

After 10-round cross-validation, we acquired the lambda with the minimum partial likelihood deviance to identify genes with significant contribution to the model. With 10-round cross-validation to establish the risk model, a formula for calculating risk scores of each patient was established after being weighted by each estimated regression coefficients. The formula is as below:

Risk Score = expression of *HTRA1* × (−0.349596300606235) + expression of RBBP7 × (−0.230030729846002) + expression of NFE2L2 × (−0.172501026570002) + expression of SCAF11 × (−0.0178575107829167) + expression of ABL1 × 0.0483720222894683 + expression of PAK2 × 0.14510870902176 + expression of CPTP × 0.159731292856169 + expression of ADORA3 × 0.213698446606236.

To validate the performance of the risk prediction model, we tested the performance of risk model in internal validation set (the residual 20% of GSE10846) and external validation datasets (TCGA datasets and GSE4475). The time-dependent receiver-operating characteristic (ROC) was conducted via R ‘survivalROC’ package to assess the accuracy of model predictions.

### 2.4. Construction of Nomogram

The nomogram was designed to predict survival of DLBCL patients. Risk score, age, gender, stage, ECOG status, lactate dehydrogenase (LDH) ratio, and extra nodalsites were used to build the nomogram using R “rms” and “survival” packages. For nomogram test, proportional hazard assumption was conducted through Schoenfeld residual calculation.

### 2.5. Immune Components Prediction

“GSVA” R package [17] (version 1.36.2) was applied to calculate single-sample GSEA (ssGSEA) scores of each immune cell infiltration and function in each sample. ssGSEA were performed with parameters as method = ‘ssgsea’, kcdf = ‘Gaussian’.

### 2.6. Prediction of Chemosensitivity

Based on the largest publicly available pharmacogenomics database (GDSC, the Genomics of Drug Sensitivity in Cancer, https://www.cancerrxgene.org/ (accessed on 10 October 2022)), we predicted the chemotherapeutic sensitivity for the high-risk group and low-risk group via R package “pRRophetic”. The estimated IC50 of each treated with specific chemotherapy drug was obtained by ridge regression, and prediction accuracy was measured through 10-fold cross-validation with the GDSC training set. Default values were set for all parameters, including ‘combat’ to average replicate gene expression levels after removing batch effects.

### 2.7. Cell Culture

Germinal center B-like (GCB) DLBCL cell lines used in this study were OCI-Ly-1, Karpas, DB, SUDHL-4, SUDHL-6, SUDHL-8, and SUDHL-10. Activated B cell-like (ABC) DLBCL cell lines used were HBL-1, OCI-Ly-3, OCI-Ly-10, and SUDHL-2. Cells were obtained from Shanghai Institute of Hematology (Ruijin Hospital affiliated to School of Medicine, Shanghai Jiao Tong University, Shanghai, China) and Department of Biochemistry and Molecular Cell Biology, Shanghai Jiao Tong University School of Medicine, Shanghai, China. Briefly, cells were cultured in Gibco/BRL RPMI-1640 medium (Gibco, New York, NY, USA) added with 10% FBS (Gibco, New York, NY, USA) under a 5% CO_2_ humidified atmosphere environment at 37 °C. OCI-Ly-1, OCI-Ly-3 and OCI-Ly-10 cell lines were respectively cultured in IMDM medium (Gibco, New York, NY, USA) supplemented with 10% FBS.

### 2.8. RT-qPCR

Total RNAs were extracted from different cell line samples with TRIzol reagent (Invitrogen, Carlsbad, CA, USA). cDNA template was then synthesized using a PrimeScript RT Kit (Takara Bio Inc., Beijing, China). RT-qPCR was conducted using a SYBR qPCR Master Mix (vazyme, Q711-02) on a Roche LightCycler^®^ 480 machine. All primer sequences were listed in Table 1.

### 2.9. Statistical Analysis

Bioinformatic and statistical analysis was conducted with R software (ver. 4.0.5) and GraphPad Prism software (ver. 9.0.0.121) in this study. Data were displayed as the mean and standard error of measurement (SEM). A Kaplan-Meier method and log-rank comparation was performed for survival rate assessment. Multivariate analysis was developed based on a cox proportional hazards model. Bi-directional detection, Pearson’s correlations and Spearman’s correlations were calculated using ‘Cor.test’ functions with R software. Statistical tests in this study were all two-sided and *p* < 0.05 was considered statistically significant.

## 3. Results

### 3.1. Identification of 24 Prognostic Pyroptosis Related Genes (PRGs) in DLBCL

**Figure 1:**
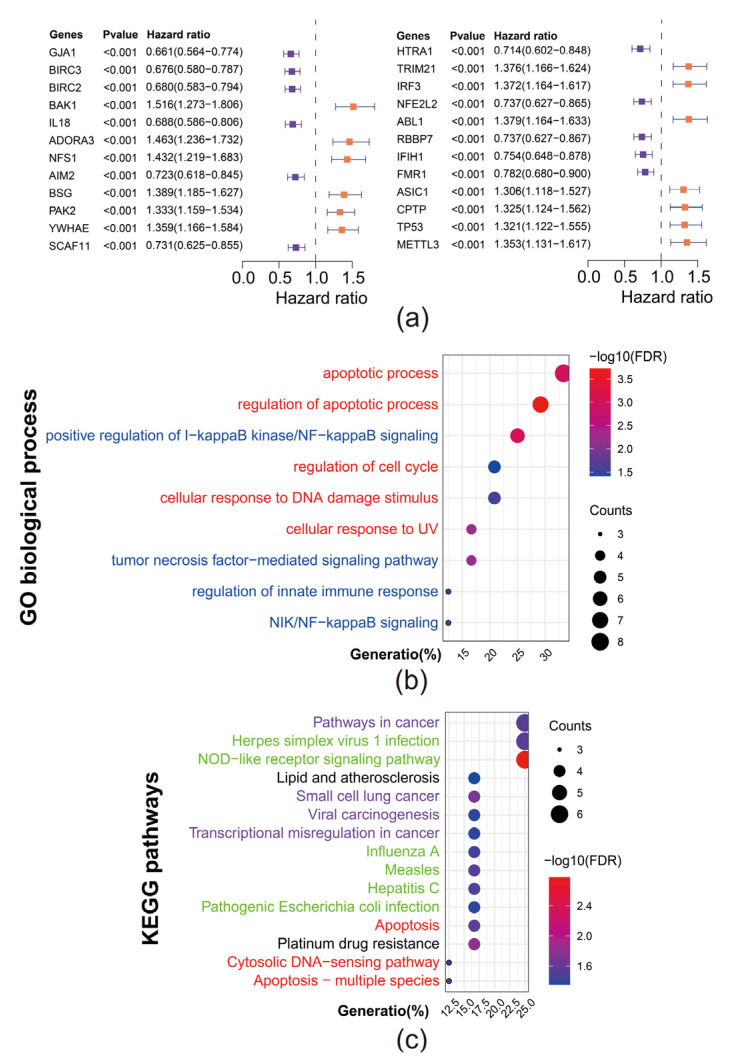
Identification of 24 prognostic pyroptosis related genes (PRGs) in DLBCL. (**a**) Univariate Cox regression identified 24 prognostic PRGs in DLBC; (**b**) Gene ontology annotation of 24 prognostic PRGs; (**c**) KEGG pathway enrichment analysis of 24 prognostic PRGs.

To investigate the role of pyroptosis in the progression of DLBCL, we conducted a univariate COX regression of 388 obtained PRGs to screen out the protective and hazard PRGs in 412 patients from GSE10846. Genes with *p* < 0.001 were considered with higher confidence and selected to carry the further analysis (Figure 1a). Among those 24 genes, genes with hazard ratio >1 were considered as hazard PRGs (in orange) whereas those with hazard ratio <1 were considered as protective PRGs (in purple). These protective or hazard genes collectively affect the prognosis of DLBCL.

To further investigate the biological function of these prognostic PRGs, we conducted pathway enrichment analysis with Gene Ontology biological process and KEGG database from DAVID to annotate these genes (Figure 1b,c). To ensure the confidence of analysis, we selected terms whose FDR < 0.05 to present. The full list of pathway terms is available in Supplementary Table S3. The results of Gene Ontology biological process annotation indicated that these genes mainly engaged in two processes: (1) apoptosis related process (in red) and (2) inflammation related process (in blue). Meanwhile, the results of KEGG annotation, in line with the results of Gene Ontology biological process, can be divided in four categories: (1) Cancer related pathways (in purple); (2) Pathogenic infection mediated pathways (in green); (3) Apoptosis related pathways (in red) and (4) Other pathways (in black).

### 3.2. Establishment of a PRGs Oriented Risk Model via LASSO Regression

**Figure 2:**
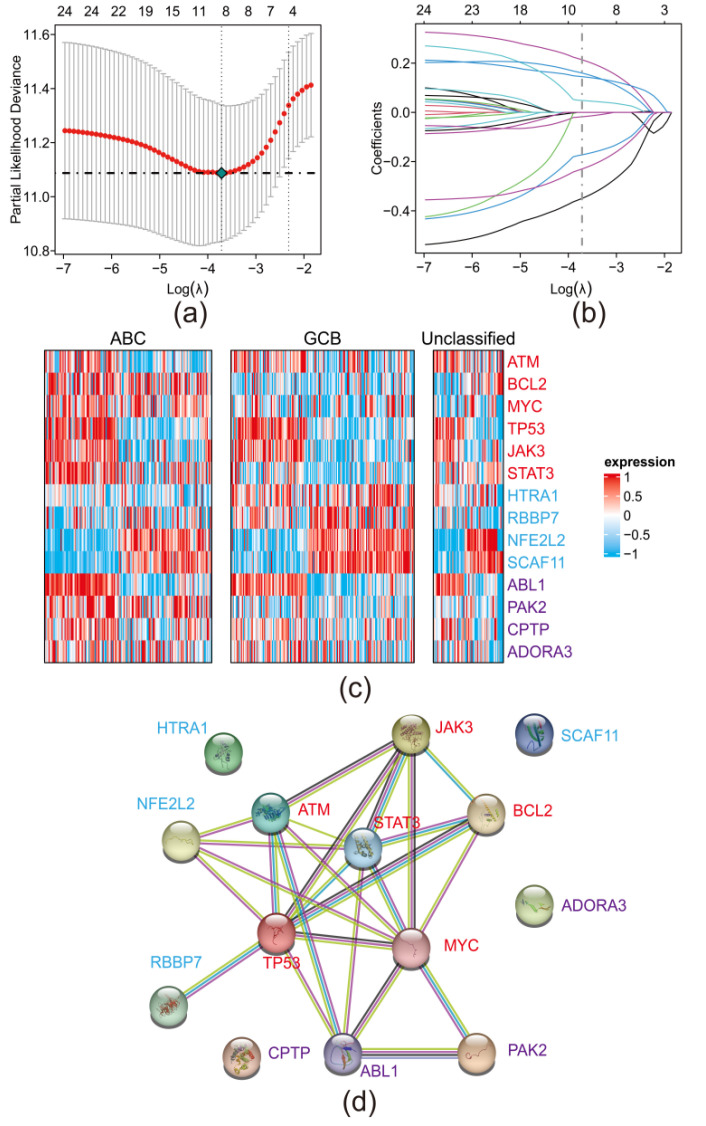
Establishment of a PRGs oriented risk model via LASSO regression: (**a**) Identification of minimum lambda via cross validation; (**b**) Identification of genes with significant contribution to the model and corresponding coefficients; (**c**) Heatmap of onco-genes, 4 protective PRGs and 4 hazard PRGs in patients with different molecular subtype of DLBCL; (**d**) Protein–protein interaction network of onco-genes, 4 protective PRGs and 4 hazard PRGs; blue lines suggested a known interaction from curated databases; purple lines suggested a known interaction that was experimentally determined; yellow lines suggested that the interaction information was from text mining; black lines suggested that the type of interaction was co-expression.

On the foundation of 24 prognostic PRGs, we established a risk model to anticipate the risk and prognosis of DLBCL. First, we randomly selected 80% of patients from GSE10846 as the training set for our LASSO regression model. After 10-round cross-vali dation, we acquired the lambda with the minimum partial likelihood deviance (Figure 2a) to identify genes with significant contribution to the model (Figure 2b). Finally, a model comprising 8 PRGs (*HTRA1*, *RBBP7*, *NFE2L2*, *SCAF11*, *ABL1*, *PAK2*, *CPTP,* and *ADORA3*) was established. The coefficient corresponding to each gene and the formula to generate risk score are available in Materials and Methods section. The coefficients of each genes suggested that *HTRA1*, *RBBP7*, *NFE2L2,* and *SCAF11* were protective whereas *ABL1*, *PAK2*, *CPTP,* and *ADORA3* were hazardous.

Based on the molecular subtypes of DLBCL, patients in GSE10846 can be divided into three categories: Germinal center B-like (GCB), Activated B cell-like (ABC) and unclassified. By heatmap (Figure 2c), we found that some onco-genes, especially *BCL2* and *MYC*, were highly expressed in ABC patients and two protective PRGs (*HTRA1* and *RBBP7*) were highly expressed in GCB patients. In general, there is no significant expression pattern of PRGs in three different molecular subtypes of DLBCL.

Further, the protein–protein interaction network analysis revealed a complex interaction between oncogenes, protective PRGs, and hazard PRGs (Figure 2d).

### 3.3. Risk Model Performed Sensitively and Robust in Internal and External Validation Datasets

**Figure 3:**
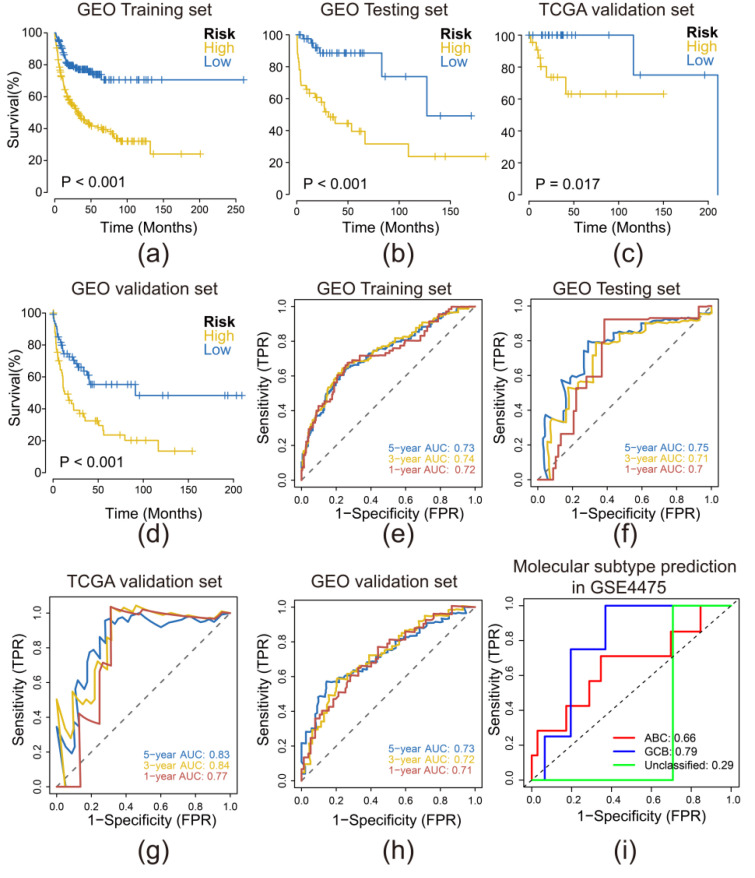
Risk model performed sensitively and robust in Internal and external validation datasets (**a**–**d**) K-M survival plots of high- and low-risk groups in training, testing, and validation datasets (**e**–**h**) ROC curve and AUC of the risk model in training, testing, and validation datasets (**i**) ROC curve and AUC of molecular subtype prediction of the risk model in GSE4475.

To validate the performance of our risk model, we conducted Kaplan–Meier survival analysis and time-dependent receiver-operating characteristic (ROC) analysis. First, we generated risk score corresponding to each patient and subsequently divided patients into the high-risk set and low-risk set based on the median risk score. Then survival analysis was performed on these patients in training set (80% of GSE10846), internal testing set (residual 20% of GSE10846), and external validation datasets (GSE4475 and TCGA) (Figure 3a–d). The results of survival analysis in these four datasets all indicated that patients at high risk depicted a significantly worse prognosis.

We conducted a time-dependent receiver-operating characteristic (ROC) analysis to assess the accuracy of risk model (Figure 3e–h). The area under the curve (AUC) represents the accuracy of the anticipation. High AUC suggested a high accuracy. The AUCs of the model in four datasets were higher than 0.7, suggesting that our risk model is a preferable, sensitive, and robust prediction model. Further, our model presented a high AUC of predicting GCB type of DLBCL (Figure 3i)

In comparison, we conducted Ridge regression to generate a risk model under similar procedure (Figure S1). The Ridge regression model contained more variables than LASSO regression model and performed a similar AUC in GSE10846 datasets, but a decreased AUC in external (GSE4475 and TCGA) datasets. Overall, LASSO regression model performed better.

### 3.4. RT-qPCR and IHC Slice Image Confirmed the Expression of 8 PRGs in Real World

**Figure 4:**
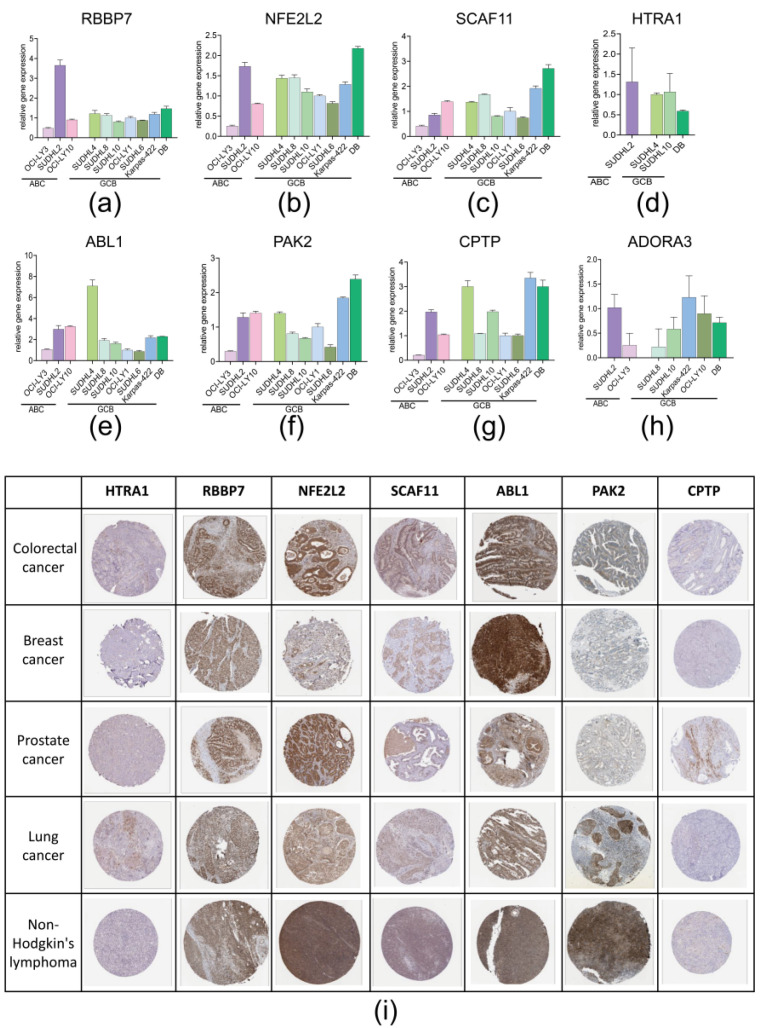
RT-qPCR and IHC slice image confirmed the expression of 8 PRGs in real world: (**a**–**h**) RT-PCR validation of 8 PRGs in vitro ABC and GCB cell lines; (**i**) IHC images of 7 PRGs in colorectal cancer, breast cancer, prostate cancer, lung cancer, and Non-Hodgkin’s Lymphoma.

We previous validated the performance of model in silico and confirmed the robust of this model. To ensure the expression of 8 PRGs in DLBCL, we assessed the expression of 8 PRGs in 10 cell lines (7 GCB DLBCL cell lines and 3 ABC DLBCL cell lines) via RT-qPCR. The results showed that *RBBP7*, *NFE2L2*, *SCAF11*, *ABL1*, *PAK2,* and *CPTP* expressed in all 10 cell lines, whereas the *HTRA1* and *ADORA3* expressed in part of DLBCL cell lines (Figure 4a–h). Further, we compared the expression of 8 PRGs in ABC and GCB cell lines. Results indicated no significant difference between two type of cell lines (Figure S2A–H). An additional ABC cell line, HBL-1, was also utilized but excluded for comparison because the expression of *RBBP7*, *PAK2*, *NFE2L,2* and *SCAF11* was significantly higher, making it improper to be compared with expression in other cell lines (Figure S2I).

Beyond cell lines, we obtained the IHC slice images of *HTRA1*, *RBBP7*, *NFE2L2*, *SCAF11*, *ABL1*, *PAK2,* and *CPTP* in colorectal cancer, breast cancer, prostate cancer, lung cancer, and Non-Hodgkin’s Lymphoma from The Human Protein Atlas (HPA) database to assess the expression of these genes in human tumor tissues, although the IHC image of *ADORA3* was not available. *HTRA1* and *CPTP* showed a weak expression whereas the other five genes were potently stained. Collectively, these 8 PRGs were confirmed to express in the real world.

### 3.5. The Risk Score Predicted the Advance of DLBCL

**Figure 5:**
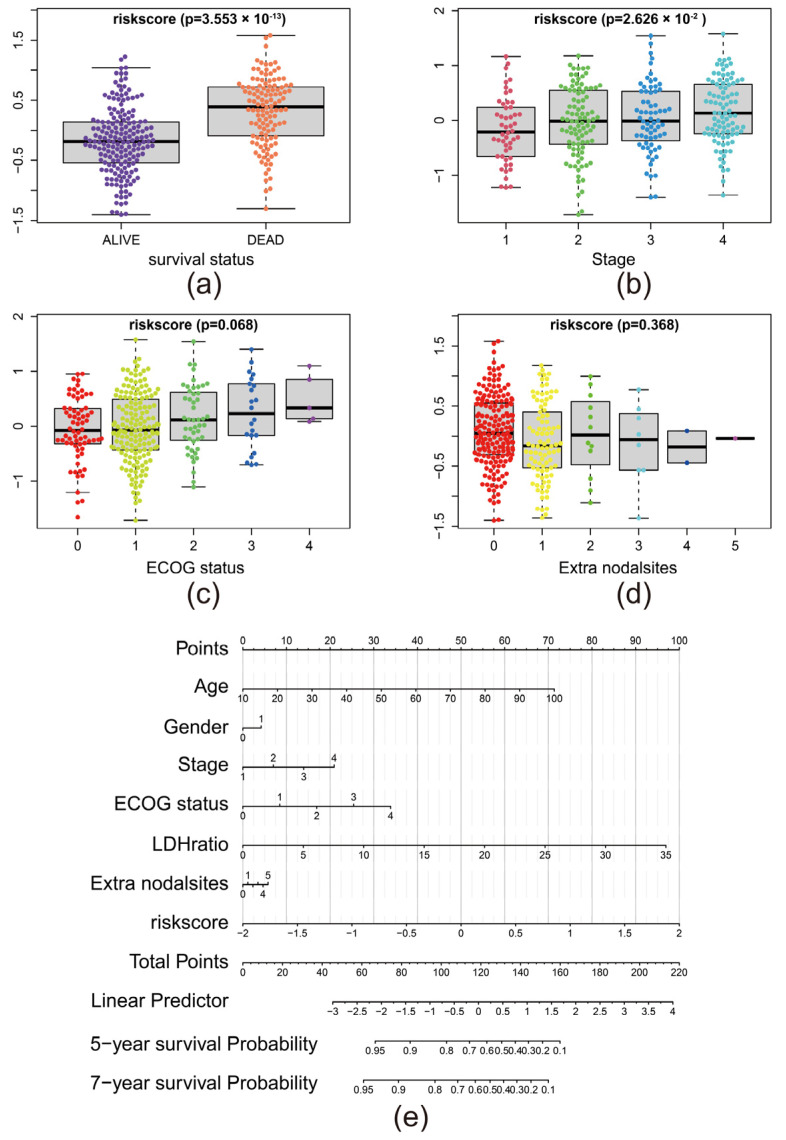
The risk score predicted the advance of DLBCL: (**a**–**d**) Risk score distribution in different survival, stage, ECOG status, extra nodal sites group; (**e**) Nomogram of the risk model based on age, gender, stage, ECOG status, LDH ratio, extra nodal sites, and risk score. In Gender, item 0 stands for male, and item 1 stands for female.

We explored the relationship between risk score and survival status, clinical stages, Eastern Cooperative Oncology Group (ECOG) status, and Extra nodal sites (Figure 5a–d). Clinical stages reflect the advance of DLBCL. ECOG status is the physical activity index of the patient. The higher the ECOG status is, the worse the patient’s physical status is. The results indicated that higher risk score was related to an advanced stage. Notably, higher risk scores may also relate to an advanced ECOG status with a *p* close to 0.05.

To extend the usage of our risk in clinical practice, we performed nomogram analysis (Figure 5e). We combined several vital and basic variables (age, stage, ECOG status, lactate dehydrogenase (LDH) ratio, and extra nodal sites) and risk scores of each patient to comprehensively evaluate the survival probability. Meanwhile, we validated the performance of nomogram in anticipating five-year and seven-year survival probability of patients with DLBCL via Calibration comparison (Figure S3A). The results suggested that our model is stable in anticipating five-year and seven-year survival probability.

### 3.6. The Risk Model Predicted the Sensitivity of Novel Chemotherapy

**Figure 6:**
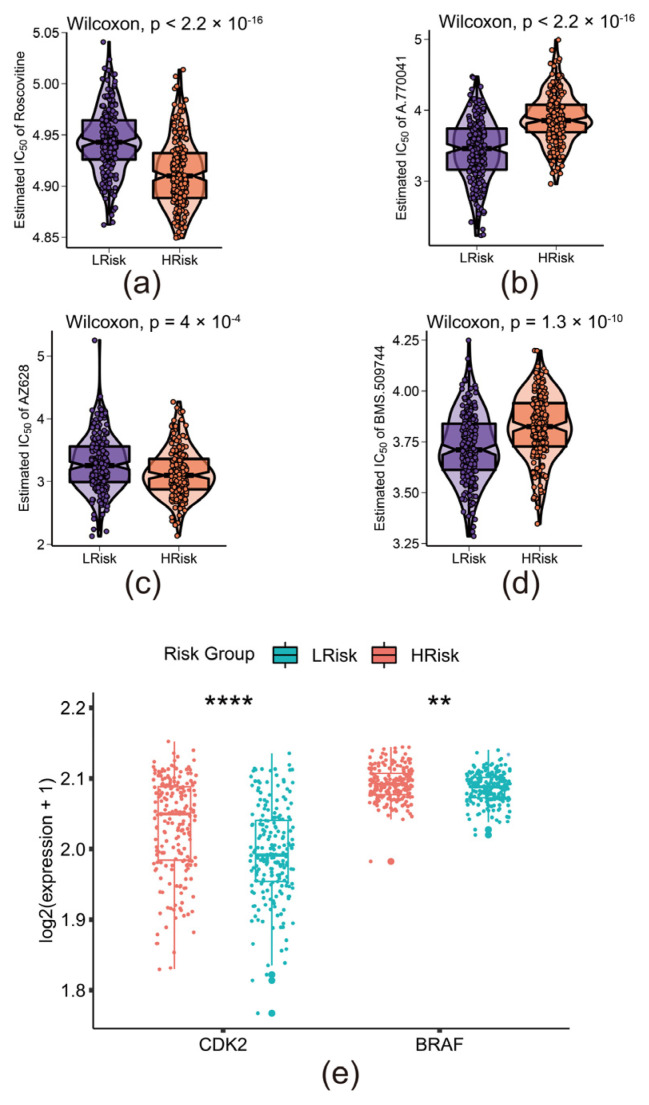
The risk model predicted the sensitivity of novel chemotherapy: (**a**–**d**) Prediction of chemotherapy sensitivity in high- and low-risk groups based on estimated IC50; LRisk stands for low-risk group; HRisk stands for high-risk group; (**e**) Distributions of targets of two sensitive chemotherapy in the high-risk group; **: *p* < 0.01; ****: *p* < 0.0001.

To find novel potential chemotherapy drugs, we predicted the chemotherapeutic sensitivity for the high-risk group and the low-risk group via R package “pRRophetic” (Figure 6 a–d). We screened out two sensitive chemotherapies (Roscovitine and AZ628) for patients with high-risk scores and two other sensitive chemotherapies (A.770041 and BMS.509744) for patients with low-risk score. We also found that *CDK2* (target of Roscovitine) and *BRAF* (target of AZ628) were highly expressed in high-risk group (Figure 6e).

### 3.7. The Risk Model Revealed the Remodeling of Immune Landscape in DLBCL

**Figure 7:**
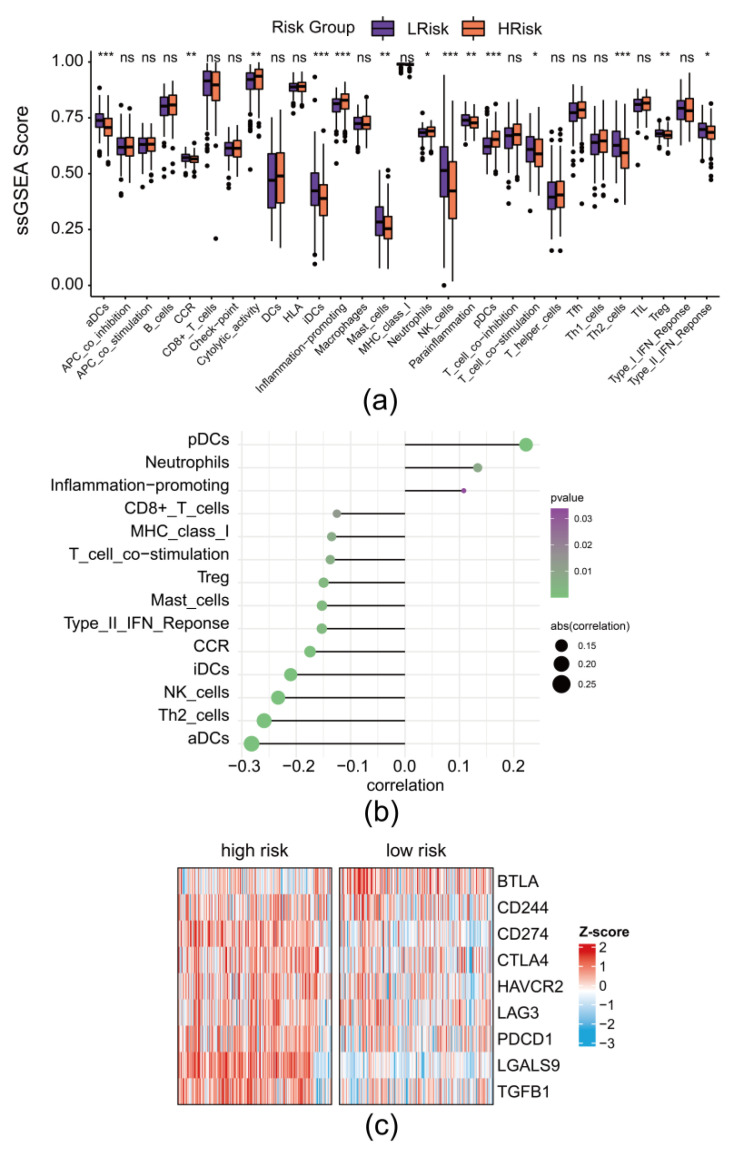
The risk model revealed the remodeling of immune landscape in DLBCL: (**a**) Comparison of immune components in DLBCL between the low-risk and high-risk groups; aDC stands for activating Dendritic Cells; iDCs stands for immature Dendritic Cells; pDCs stands for plasmacytoid Dendritic Cells; Tfh stands for Follicular helper T cell; CCR stands for Chemokine receptors; TIL stands for Tumor Infiltrating Lymphocytes; Treg stands for regulatory T cells; *: *p* < 0.05; **: *p* < 0.01; ***: *p* < 0.001; (**b**) Correlation between immune components in DLBCL and high-risk scores; (**c**) Heatmap of co-inhibitory factors in DLBCL between the low-risk and high-risk groups.

Pyroptosis was considered to be an associated inflammatory process in the tumor. We calculated single-sample GSEA (ssGSEA) scores of each immune cell infiltration and function in each sample and analyzed the difference of cell infiltration and function between high- and low-risk groups. As shown in Figure 7a, patients in the high-risk group showed a pro-inflammatory landscape but with a low adaptive immune response. The correlation between risk score and scores of each immune components further highlighted the negative association between risk score and adaptive cellular immune (Figure 7b). In addition, inhibitory immune check points were found to highly expressed in high-risk groups, suggesting an exhaustion of tumor infiltration lymphocytes (Figure 7c).

## 4. Discussion

DLBCL is an extremely heterogenous lymphoid neoplasm of B cells with a variety of clinical, pathological, and biological phenotypes [18]. Patients with DLBCL showed variations in clinical outcome due to the genetic alterations of DLBCL. Understanding the biological characteristics and genetic landscape of DLBCL is essential for the classification of DLBCL patients, and thus a more precise classification method is required to conduct personalized clinical course. Recent research [19,20,21,22] has illustrated that pyroptosis engaged in various solid carcinomas, like hepatocellular carcinoma [19] and breast cancer [20], while the role of pyroptosis in progression of DLBCL has yet to be claimed. Currently, pyroptosis has been considered as a novel potential strategy for the treatment of malignant tumors [23] and demonstrated to have prognostic value in DLBCL [15]. Thus, a comprehensive review on characteristics of multiple PRGs might provide deeper insights into regulatory mechanisms underlying progression of DLBCL and a new strategy for the diagnosis and targeted treatment of DLBCL. Hence, we established a risk prediction model from the sight of pyroptosis to predict the prognosis of DLBCL and guide the treatment of patients at high risk.

Previous studies have discovered the immune regulatory function of pyroptosis in tumor immunity [14] and wide engagement of pyroptosis in cancerogenesis and progression of malignant tumors [11]. In our study, we identified 24 prognostic PRGs in DLBCL. The enrichment analysis of GO and KEGG pathways of these PRGs were found to generally fall into three categories: (1) apoptosis related processes; (2) inflammatory pathways; (3) cancerogenesis related pathways. Such results revealed the apoptotic and inflammatory nature of pyroptosis in DLBCL and indicated the engagement of pyroptosis in progression of DLBCL. In addition, pathogenic infection mediated pathways, comprising Influenza A, Hepatitis C, and Measles etc. were enriched, suggesting that adaptive cellular immune was inhibited in DLBCL.

Based on 24 prognostic PRGs, we further screened out 8 PRGs that contributed most to establish our risk prediction model via LASSO regression. We identified four protective PRGs (*HTRA1*, *RBBP7*, *NFE2L2,* and *SCAF11*) and four hazardous PRGs (*ABL1*, *PAK2*, *CPTP* and *ADORA3*) in the model. Those hazardous PRGs were highly expressed in high-risk groups together with oncogenes such as *ATM*, *BCL2*, *MYC*, *TP53*, *JAK3*, *STAT3*, *ABL1* [24,25,26,27], *PAK2* [28,29,30,31], and *ADORA3* [32] were previously reported to engage in proliferation and invasion of various solid tumor. *CPTP* was found to be involved in pyroptosis [33], and while its pro-tumor function in DLBCL was discovered in our study, *HTRA1*, *RBBP7*, *NFE2L2* and *SCAF11* were found to be tumor suppressive. Further, our protein–protein interaction network suggested a complex interaction between oncogenes, protective, and hazard PRGs. These PRGs may act in an anti-tumor or pro-tumor role in DLBCL via interactions with oncogenes. After the establishment of the model, we validated our model in silico and in vitro. The AUC values of ROC curve were applied to assess the accuracy of the model. The AUC values of one-, three-, and five-years were all higher than 0.7 in four datasets, suggesting a robust performance in prediction. Intriguingly, our model suggested to have power in classifying the GCB and non-GCB subtype of DLBCL.

Further, we assessed mRNA expression of these eight PRGs in seven GCB DLBCL cell lines and three ABC DLBCL cell lines via RT-qPCR. The IHC images obtained from HPA indicated that these seven PRGs (except ADORA3) expressed in human colorectal cancer, breast cancer, prostate cancer, lung cancer, and Non-Hodgkin’s Lymphoma. Collectively, we confirmed the expression of these eight PRGs in real word. The expression of these PRGs in the real world laid a solid foundation for the use of model in clinical practice.

Then, we investigated the correlation between risk score, survival status, clinical stages, ECOG status, and Extra nodal sites. We found that a high risk score is associated with advanced clinical stages and ECOG status. An advanced clinical stage suggested the progression and metastasis of tumor. Patients with high ECOG status have a weak physical status. Patients with a combination of high ECOG status and advanced clinical stages were unsuitable for surgery, chemotherapy, and radiotherapy. To comprehensively evaluate the survival probability, we designed a nomogram with age, gender, stage, ECOG status, LDH ratio, and extra nodal sites and risk scores. From this nomogram, we were able to predict the five-, and seven-year survival of DLBCL, extending the use of the model in clinical practice. Beyond prediction of prognosis, we screened out two novel chemotherapies with low estimated IC50 for high-risk patients. Roscovitine is one of the members of the CDK-inhibitor family, primarily inhibiting CDK2 to restrict the proliferation of cancer cells [34]. AZ628, a selective RAF kinase inhibitor, inhibits BRAF-mediated cell proliferation and achieves meaningful clinical benefit [35,36]. Further, CDK2 and BRAF, targets of these two chemotherapies, were highly expressed in high-risk scores. Although these two chemotherapies were not standard treatment for DLBCL, patients in high-risk groups showed a significantly lower estimated IC50 and higher expression of *CDK2* and *BRAF*, suggesting the potential value of Roscovitine and AZ628 as a rescue therapy.

Chronic inflammation, however, suppresses the immune cellular anti-tumor responses, which are crucial for tumor initiation, progression, and invasion. We found that the scores of immune components were different in high- and low-risk groups. The score of adaptive cellular immune components, such as NK cells and aDC cells, were significantly lower in high-risk groups. In addition, inflammation related cells such as neutrophils in high-risk groups were increased. Such results indicated that pyroptosis potentially activated the innate immune and inhibited the adaptive cellular immune which is essential for anti-tumor immune. The correlation between risk score and scores of each immune component further underlined the negative association between risk score and adaptive cellular immune components. High risk score was associated with high neutrophils and low CD8 + Tcells. Further, immunosuppressive factors were found to be highly expressed in aa high-risk group, suggesting the exhaustion of tumor infiltrated lymphocytes in high-risk patients. Collectively, pyroptosis potentially remodeled the immune landscape of DLBCL.

**Figure 8:**
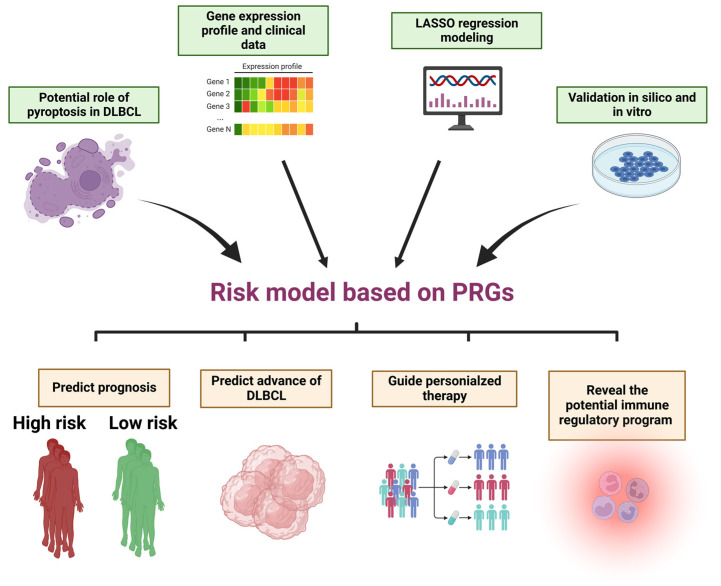
The workflow map. Created with BioRender.com (accessed on 31 October 2022). The publication license is available in Supplementary File S1.

In agreement with previous studies, our findings provided further evidence that pyroptosis played a double-edged sword of a role in DLBCL and the potential immune regulatory function in DLBCL. The novel model we established can be utilized in clinical practice to anticipate the prognosis of DLBCL and guide the treatment of patients at high risk. Notably, limitation still exists in our study. Although we validated our model in silico and in vitro, more cohorts of real-world patients are required to modify this model. Additionally, further experiments are also required to verify the mechanism underlying the modulation of immune landscape in DLBCL via pyroptosis (Figure 8).

## 5. Conclusions

All in all, our work established a model that can be utilized in clinical practice to predict the prognosis of DLBCL and guide the treatment of patients in high risk, and provide an overview of immune regulatory program via pyroptosis in DLBCL.

## Figures and Tables

**Table 1:** Primer sequences of 8 pyroptosis related genes.

Genes	Forward Primer (5′-3′)	Reverse Primer (5′-3′)
HTRA1	TCCCAACAGTTTGCGCCATAA	CCGGCACCTCTCGTTTAGAAA
RBBP7	GAGGAGCGTGTCATCAATGAA	GCATGGGTCATAACCAGGTCATA
NFE2L2	TTCCCGGTCACATCGAGAG	TCCTGTTGCATACCGTCTAAATC
SCAF11	TGAAAGCAAAGTGTACCAACCT	GGCTCTCTATAAGCTCCTCTGT
ABL1	TGAAAAGCTCCGGGTCTTAGG	TTGACTGGCGTGATGTAGTTG
PAK2	CACCCGCAGTAGTGACAGAG	GGGTCAATTACAGACCGTGTG
CPTP	TCTGCGCCGACTCCTACAA	CTCGCCTAGCATCTGCACG
ADORA3	GGCCAATGTTACCTACATCACC	CCAGGGCTAGAGAGACAATGAA

## Data Availability

Publicly available datasets were analyzed in this study. The data can be found here: GSE10846 and GSE4475 dataset at https://www.ncbi.nlm.nih.gov/geo/ (accessed on 10 September 2022), TCGA dataset at The Cancer Genome Atlas (TCGA) database. The IHC images can be assessed at The Human Protein Alta (HPA). Gene sets used for ssGSEA can be found in the Supplementary Materials of article generated by Xiao B et al. [16].

## Supplementary Materials

The following supporting information can be downloaded at: https://www.mdpi.com/article/10.3390/biom12121835/s1, Figure S1: Establishment and performance of Ridge regression model; Figure S2: Comparison of expression of 8 PRGs in ABC and GBC cell lines; Figure S3: Calibration comparison of nomogram; Table S1: List of pyroptosis related genes obtained from GeneCards.xlsx; Table S2: Results of Cox univariate regression of 24 prognostic pyroptosis related genes.xlsx; Table S3: List of GO pathway enrichment.txt; Table S4: List of KEGG pathway enrichment.txt; Table S5: Coefficient of genes in lasso regression model.xlsx; Table S6: Patients id of IHC images obtained from HPA database.xlsx; Table S7: Results of protein-protein interaction between 8 PRGs and master genes of DLBCL.xlsx; Table S8: Gene list of immune components for ssGSEA analysis.xlsx. Supplementary File S1: Publication License of Figure 8.
