# Supplementary material for: A Novel and Validated 8-Pyroptosis-Related Genes Based Risk Prediction Model for Diffuse Large B Cell Lymphoma
Source: Biomolecules. 2022 Dec 8;12(12):1835. doi: 10.3390/biom12121835 (PMC9775483; doi:10.3390/biom12121835)

A Novel and Validated 8- Pyroptosis-Related Genes Based Risk Prediction Model for Diffuse Large B Cell Lymphoma

Figure S1 Establishment and performence of Ridge regression model
A Identification of minimum lambda via cross validation
B Identification of genes with significant contribution to the model and corresponding coefficients
C The variables of Rigde regression model
D-G ROC curve of Ridge regression model in training, testing and validation datasets

Figrue S2 Comparsion of expression of 8 PRGs in ABC and GBC cell lines
A-H Comparsion of expression of 8 PRGs in vitro ABC and GCB cell lines
I Expression of 8 PRGs in HBL-1 ABC cell lines

Figure S3 Calibration comparison of nomogram
A Calibration comparison of nomogram

Supplementary Figure S1

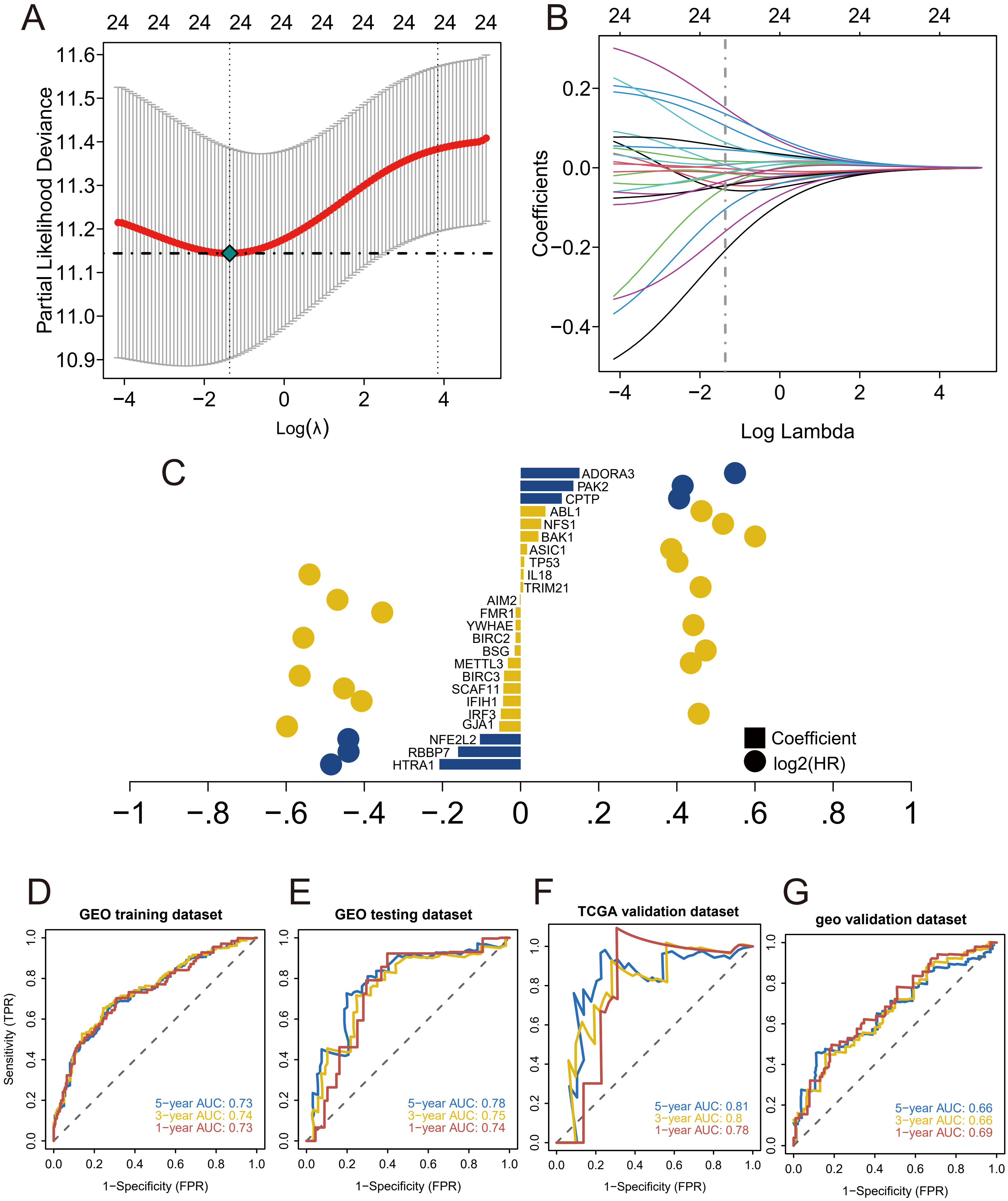

Supplementary Figure S2

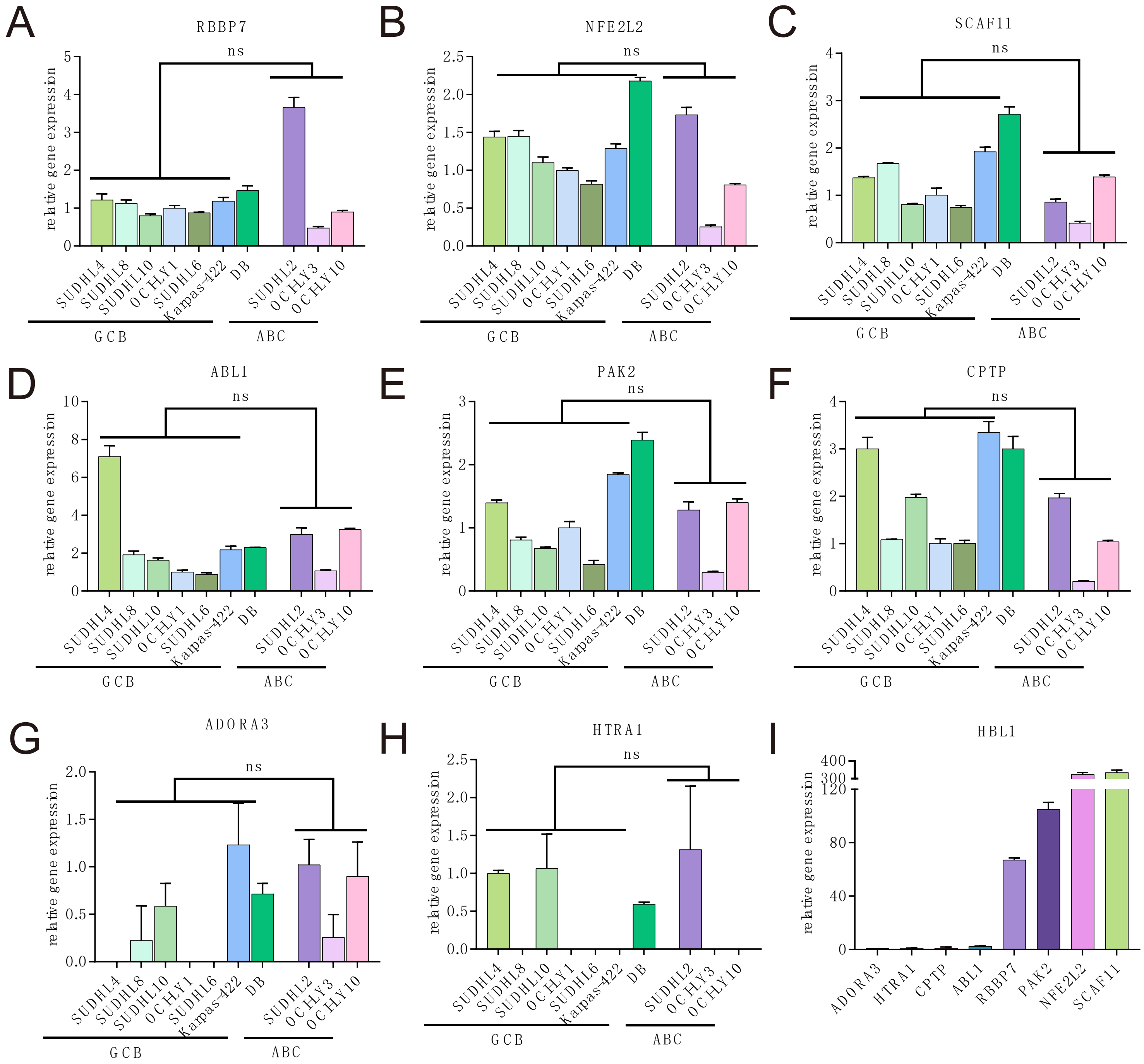

Supplementary Figure S3

A

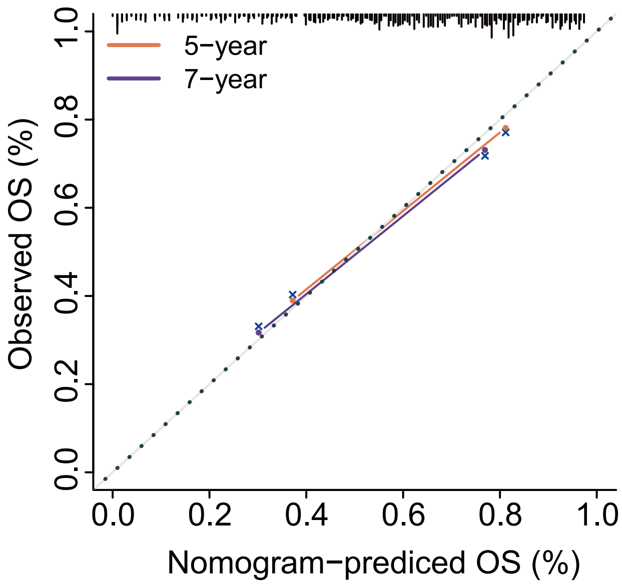

Supplement: Supplementary file 1 [file biomolecules-12-01835-s001.zip › Supplementary figures/Supplementary figure.pdf]
